# Single-nucleotide polymorphisms in Orai1 associated with atopic dermatitis inhibit protein turnover, decrease calcium entry and disrupt calcium-dependent gene expression

**DOI:** 10.1093/hmg/ddz223

**Published:** 2019-10-10

**Authors:** Yi-Chun Yeh, Yu-Ping Lin, Holger Kramer, Anant B Parekh

**Affiliations:** 1 Department of Physiology, Anatomy and Genetics, Parks Road, Oxford, OX1 3PT UK; 2 MRC London Institute of Medical Sciences, Imperial College London, UK

## Abstract

Loss-of function mutations in Orai1 Ca^2+^ channels lead to a form of severe combined immunodeficiency, auto-immunity, muscle hypotonia and defects in dental enamel production and sweat gland function. Two single-nucleotide polymorphisms (SNPs) in Orai1 have been found and localize to the second extracellular loop. These polymorphisms associate with atopic dermatitis but how they affect Ca^2+^ signalling and cell function is unknown. Here, we find that Orai1–SNPs turnover considerably more slowly than wild type Orai1 and are more abundantly expressed in the plasma membrane. We show a central role for flotillin in the endocytotic recycling of Orai1 channels and that endocytosed wild type Orai1 is trafficked to Rab 7-positive late endosomes for lysosomal degradation. Orai1–SNPs escape the degradation pathway and instead enter Rab 11-positive recycling endosomes, where they are returned to the surface membrane through Arf6-dependent exocytosis. We find that Orai1–SNPs escape late endosomes through endosomal pH regulation of interaction between the channel and flotillin. We identify a pH-sensitive electrostatic interaction between positively charged arginine in extracellular loop 2 (K210) and a negatively charged aspartate (D112) in extracellular loop 1 that helps determine Orai1 turnover. The increase in membrane Orai1–SNP leads to a mis-match in Orai1–STIM stoichiometry, resulting in inhibition of Ca^2+^ entry and Ca^2+^-dependent gene expression. Our results identify new strategies for targeting atopic dermatitis.

## Introduction

Store-operated Ca^2+^ entry generates cytosolic Ca^2+^ signals that control diverse cellular functions, such as exocytosis, energy production, gene expression and growth and differentiation (29). Store-operated channels activate following depletion of the endoplasmic reticulum (ER) Ca^2+^ store. Physiologically, this occurs upon stimulation of either G-protein or tyrosine kinase coupled cell-surface receptors that generate the second messenger inositol trisphosphate, which then releases Ca^2+^ from the ER (34). Two key protein components of store-operated Ca^2+^ entry are the ER membrane spanning Ca^2+^ sensors STIM1 and STIM2 and the Orai Ca^2+^ channels in the plasma membrane (33). Store depletion leads to Ca^2+^ dissociation from the EF hands on STIM1 and STIM2, resulting in conformational changes that enable STIM oligomers to migrate to ER-PM junctions where they bind and open Orai channels.

Autosomal recessive null mutations in Orai1 cause a combined immunodeficiency syndrome that presents as susceptibility to recurrent viral, bacterial and fungal infections (18). Additionally, patients exhibit congenital muscular hypotonia, amelogenesis imperfecta and anhidrosis. Two types of loss-of-function mutations in Orai1 have been described to date (18); one set (A88S–Orai1, A103E–Orai1 and H165P–Orai1) abolishes protein expression whereas another (R91W–Orai1) results in a channel that can no longer be activated by STIM1. A few autosomal dominant gain-of-function mutations in Orai1 have also been found and result either in constitutive Ca^2+^ influx in the absence of store depletion (G98S–Orai1 and L138F–Orai1) or enhanced channel activity by a reduction in Ca^2+^-dependent slow inactivation of the channels (P245L–Orai1;(24)). These mutations cause tubular associated myopathy and Stormorken syndrome.

Two single-nucleotide polymorphisms (SNPs) in human Orai1 have been found and both localize to the second extracellular loop of the channel. One variant, with a frequency of heterozygosity in 306 individuals from different ethnic groups of ~ 20%, encodes a serine-to-glycine substitution at residue 218 (S218G–Orai1; NCBI SNP database rs3741596). The other SNP, which has a corresponding frequency of ~ 6%, arises from an arginine-to-serine substitution at residue 223 (N223S–Orai1; NCBI SNP database rs75603737). How these Orai1–SNPs affect channel function and downstream Ca^2+^-dependent signalling is unknown. S218G–Orai1 is significantly associated with atopic dermatitis in the Japanese population (4). The development of atopic dermatitis involves compromise of the epidermis barrier and dysregulation of the local immune system (2). Orai1 SNPs could, therefore, alter growth of keratinocytes and thereby the barrier function of skin whilst simultaneously impacting immune cell function. Understanding how Orai1–SNPs alter Ca^2+^ signalling is, therefore, of therapeutic relevance.

In this study, we find that Orai1–SNPs turnover considerably more slowly than wild type (WT) Orai1 and are more abundantly expressed in the plasma membrane. We identify a central role for flotillin in the recycling process for Orai1 channels and show that Orai1–SNPs disembark early from the cycle to escape degradation in the late endosomes and lysosomes. Our results suggest that an electrostatic interaction between extracellular loops 1 and 2 contributes to Orai1 channel recycling and open up the possibility for therapies targeted to treat diseases like atopic dermatitis.

## Results

### Orai1–SNP proteins are more abundantly expressed

**Figure 1:**
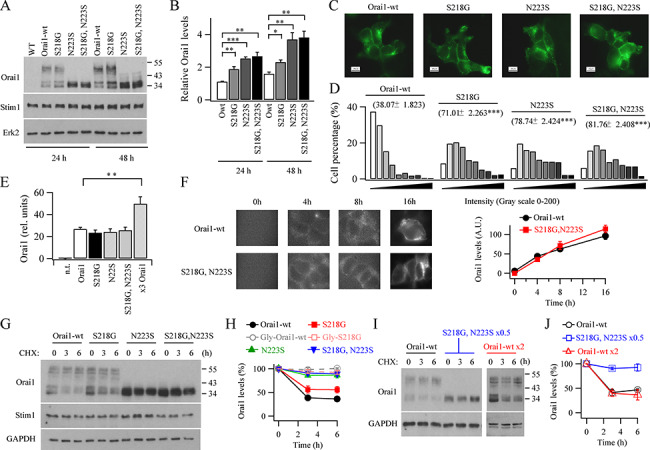
Orai1–SNP proteins are strongly expressed. (**A**) Western blot compares Orai1 WT and Orai1–SNP expression at different times after transfection. (**B**) Bar chart compares expression of the various Orai1 proteins from western blot data as in panel A. (**C**) Total cell fluorescence of GFP-tagged Orai1 proteins are compared. Data were obtained 24 hours after transfection. Each panel represents between 335 and 398 cells. (**D**) Aggregate data showing fluorescence of GFP-tagged Orai proteins are compared. Intensity (*x*-axis) is plotted in bins of 10. Mean fluorescence for each protein is at the top of each bar chart. (**E**) Quantitative PCR measurements of mRNA levels for the Orai1s shown. N.t. denotes non-transfected cells. Orai1 denotes transfection with 200 ng Orai1 plasmid. All groups were significantly different from the n.t. group (*P* < 0.001). For transfected groups, only ×3 Orai1 was significantly different from Orai1 (*P* < 0.05, one way ANOVA test). (**F**) Images compare GFP expression at different times shortly after transfection with either WT Orai1–GFP or S218G–N223S–Orai1–GFP. The graph shows the time course of expression of the two proteins. Each point is between 16 and 23 cells. There were no significant differences between corresponding data points (*P* > 0.1). (**G)** Western blot shows time course of degradation of WT Orai1 and Orai1–SNPs following treatment (0–6 hours) with cycloheximide (CHX). (**H**) Graph compares the kinetics of degradation for the various Orai1 proteins shown. At 3 and 6 hours, both Orai1-wt and S218G groups were significantly different from the others (*P* < 0.01 for each point). S218G was significantly different from Orai1-wt at 3 and 6 hours (*P* < 0.05). (**I**) The effect of either reducing the amount of Orai1–SNP expressed or increasing the amount of WT Orai1 expressed on protein degradation are compared. (**J**) Aggregate data from experiments as in panel I are compared. At 3 and 6 hours, Orai1-wt and Orai1-wtx2 were both significantly different from S218G,N223Sx0.5 (*P* < 0.01 for each point). There were no significant differences between Orai1-wt and Orai1-wtx2 (*P* > 0.1).

We compared levels of expression of WT Orai1 with the various Orai1–SNPs using western blotting, following transfection with the same amount of each plasmid. We limited the extent of overexpression by transfecting cells with only a small amount (200 ng) of Orai1 or Orai1–SNP plasmid. Endogenous Orai1 in HEK293 cells was undetectable with Sigma antibody, reflecting the low level of expression of the protein in these cells (Figure 1A). Twenty-four hours after transfection of WT Orai1, two protein bands with molecular weights around 35 kDa were observed along with a band at around 50 kDa (Figure 1A). The lower bands reflect non-glyosylated Orai1 and the upper band glycosylated protein. Transfection of S218G–Orai1, N223S–Orai1 or the double variant S218G–N223S–Orai1 plasmids all resulted in an increase in Orai1 protein levels above that seen with WT Orai1; the increase was ~ 2-fold at 24 hours and up to 3-fold at 48 hours (Figures 1A and 1B). Because N223 on Orai1 is a site for N-linked glycosylation (9), N223S–Orai1 proteins showed only the lower, non-glycosylated bands. The increase in Orai1–SNP protein expression was not accompanied by an increase in STIM1 levels (Figure 1A). Although S218G–N223S–Orai1 is not an observed human variant, we found that it expressed at consistent levels between experiments and, therefore, studied this construct in parallel with the two naturally occurring variants.

We used an immunofluorescence approach to confirm that the Orai1–SNPs were more robustly expressed at a single cell level than WT Orai1. Cells were transfected with GFP-tagged Orai1 or GFP-tagged Orai1–SNPs and then, 24 hours later, GFP fluorescence was analysed on a cell by cell basis. Grey scale absolute fluorescence intensities ranged from 5 to 194 and, therefore, were binned into intervals of 10. In cells expressing WT Orai1–GFP, most cells had low GFP fluorescence (Figure 1C) and the mean whole cell intensity was 38.1 ± 1.8 (Figure 1D). By contrast, following expression of the various Orai1–SNP–GFP constructs, most cells showed higher levels of protein fluorescence (Figures 1C and 1D). These results show that N223S–Orai1 and S218G–N223S–Orai1 protein and, to a lesser extent S218G–Orai1, are all expressed more strongly than WT Orai and with a trend that is qualitatively similar to the western blot data.

### Orai1–SNPs turnover more slowly than WT Orai1

Stronger Orai1–SNP protein expression could reflect more effective transcription, more rapid translation or that the translated protein is more stable because of reduced protein turnover. To distinguish between these possibilities, we measured Orai1 mRNA levels using quantitative PCR for each construct 24 hours after transfection. However, mRNA levels between Orai1 and Orai1–SNPs were similar (Figure 1E). We measured the rate of translation of Orai1 along with the Orai1 SNPs and the double variant by transfecting cells with GFP-tagged plasmids and then following the time course of GFP expression over the first few hours. GFP fluorescence increased with similar time courses for the various Orai1 constructs expressed over the first 8 hours and then started to diverge (Figure 1F), suggesting the initial rate of translation of each protein was similar. To measure Orai1 protein turnover, we exposed cells to the protein synthesis inhibitor cycloheximide and then measured protein expression at different times after cycloheximide treatment. For WT Orai1 protein, non-glycosylated protein expression fell by ~ 60% within 3 hours and then stabilized (Figure 1G and 1H). Non-glycosylated S218G–Orai1 protein levels also declined following cycloheximide treatment but to a lesser extent (Figures 1G and 1H). However N223S–Orai1 and S218G–N223S–Orai1 non-glycosylated protein expression were both relatively stable, declining by only ~ 15% 6 hours after cycloheximide treatment (Figures 1G and 1H). By contrast, glycosylated WT Orai1 protein (labelled Gly–Orai1–WT) and S218G–Orai1 (Gly–S218G–Orai1) were both relatively stable even in the presence of cycloheximide (Figure 1G). Since non-glycosylated Orai1 channels show a slightly larger Ca^2+^ flux (9) and turn over considerably more quickly (Figure 1), we focussed on these channels.

The difference in non-glycosylated protein turnover between WT Orai1 and S218G–N223S–Orai1 was maintained even after we reduced the expression of the latter by transfecting half the amount of DNA, to produce Orai1 double variant protein expression comparable with that of WT Orai1 (Figure 1I and 1J). Similarly, after increasing the amount of WT Orai1 expressed (~2–3-fold), protein turnover was still considerably faster than S218G–N223S–Orai1, despite both proteins exhibiting similar levels of expression immediately prior to cycloheximide exposure (Figure 1I and 1J). Therefore, reduced Orai1–SNP protein turnover is not simply due to saturation of the protein removal/degradation pathway.

### WT Orai1 is degraded by the endo-lysosomal system

To see whether the rapid loss of non-glycosylated Orai1 in the presence of cycloheximide involved lysosomal degradation, we treated cells with bafilomycin A, an inhibitor of lysosomal protein degradation through block of vacuolar H+-ATPase pump (43). Orai1 protein loss in the presence of cycloheximide was significantly reduced (Supplemental Figure 1). An alternative way to prevent acidification of intracellular compartments including lysosomes is to expose cells to an NH_4_Cl pulse. An NH_4_Cl pulse also reduced the degradation of WT Orai1 protein that occurred in the presence of cycloheximide (Supplemental Figure 1).

Protein levels of S218G–N223S–Orai1 increased slightly in the presence of bafilomycin A (Supplemental Figure 1), demonstrating that this too was degraded by the endo-lysosomal pathway, albeit to a considerably lesser extent than WT Orai1.

Orai1 has been reported to interact with ubiquilin 1(20), which leads to ubiquitination and protein degradation. However, following co-expression of Orai 1 and ubiquitin, we were unable to detect the presence of the ubiquitinylated Orai1 ladder, which is indicative of ubiquitin-mediated degradation (Supplemental Figure 1).

### Comparison of WT Orai1 and Orai1 SNP protein in the plasma membrane

**Figure 2:**
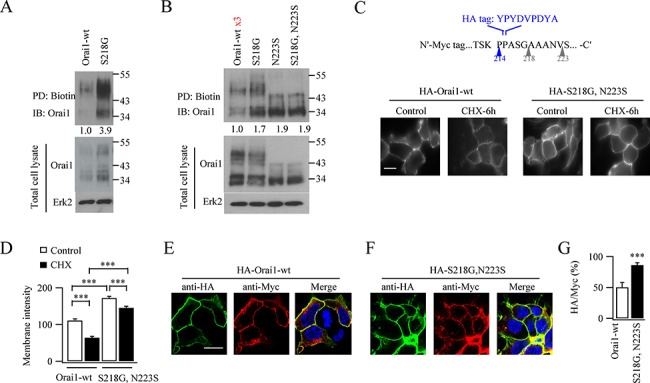
Surface expression of Orai1–SNPs is higher than WT Orai1. (**A**) Gel compares biotinylation of WT Orai1 with S218G–Orai1. Biotinylated protein was pulled down (PD) and then samples immunoblotted (IB) for Orai1 (HA-tagged). (**B)** Biotinylated Orai1–SNPs are compared with WT Orai1, the latter being expressed at ×3 the normal level to match total protein expression between the various Orai1s. Numbers below the upper gel denote the extent of biotinylation relative to WT Orai1. (**C**) Surface expression of WT Orai1 and Orai1–SNP are compared at rest and then after 6 hours exposure to cycloheximide. An HA tag was inserted into the second extracellular loop for each protein (inset). (**D**) Surface intensity of HA fluorescence is compared between WT Orai1 and Orai1–SNP, for the conditions shown. Data were obtained from experiments as in panel C. Each bar denotes between 94 and 164 cells. (**E**) Surface and cytosolic expression of WT Orai1 is shown, obtained by expressing an HA- and myc-tagged construct (see text for details). (**F**) As in panel E but S218G–N223S–Orai1 was expressed instead. (**G**) Bar chart compares relative surface expression of WT Orai1 and Orai1–SNP.

We used a biotinylation approach to measure plasmalemmal Orai1 protein levels. Following exposure to biotin, we immuno-precipitated biotinylated membrane protein and immunoblotted for Orai1. Twenty-four hours after transfection, ~ 4-fold more S218G–Orai1 was biotinylated than WT Orai1 (Figure 2A). However, this difference could be explained by the lower cellular expression of WT Orai1. We, therefore, transfected a greater amount of WT Orai1 so that expression was now similar to that for S218G–Orai1, N223S–Orai1 and S218G–N223S–Orai1. Although transfection with 3 times more WT Orai1 led to similar levels of expression between WT Orai1 protein and the two Orai1–SNPs or double variant (Figure 2B, lower gel), biotinylated WT Orai1 protein was considerably less than for S218G–Orai1, N223S–Orai1 and S218G–N223S–Orai1 (Figure 2B).

**Figure 3:**
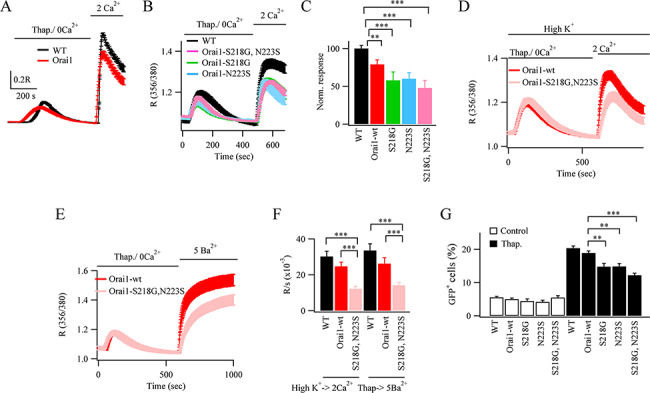
Orai1–SNPs reduce store-operated Ca^2+^ entry. (**A**) Store-operated Ca^2+^ influx is compared between mock-transfected WT cells and cells transfected with WT Orai1. Cells were stimulated with thapsigargin (2 μM) in Ca^2+^-free solution followed by readmission of 2 mM external Ca^2+^, as shown. Each trace is the mean of between 28 and 44 cells. (**B**) Ca^2+^ influx is compared between WT cells and cells expressing the various Orai1–SNPs. (**C**) Aggregate data, showing the rate of rise of cytosolic Ca^2+^ following Ca^2+^ readmission are compared. (**D**) Ca^2+^ influx is compared between WT Orai1 and Orai1–SNP when cells were bathed in high (100 mM) K^+^. (**E**) Ba^2+^ entry is compared in cells over expressing WT Orai1 or expressing S218G–N223S–Orai1. (**F**) Aggregate data from experiments as in panels D and E are compared. Bars are mean of between 18 and 30 cells. (**G**) GFP reporter gene expression is compared for the conditions shown, before and then after stimulation with thapsigargin. Each bar denotes between 295 and 337 cells.

To examine surface expression in a different way, we inserted a small HA tag into the second extracellular loop of Orai1 (inset of Figure 2C) and then used anti-HA immunofluorescence to compare surface expression of WT Orai1 with the various Orai1–SNPs. Surface fluorescence of HA-tagged S218G–N223S–Orai1 was ~ 1.7-fold higher than when WT Orai1 was expressed (Figure 2C; cells were transfected with ×3 the usual amount of WT Orai1 to ensure similar levels of protein expression). Treatment with cycloheximide for 6 hours significantly reduced surface expression of WT HA-tagged Orai1 by ~ 50% but had only a weak effect on levels of S218G–N223S–Orai1 (Figure 2D).

We estimated the relative amounts of Orai1 protein in the cell surface and cytoplasm by constructing a double-labelled Orai1 protein, with an HA tag in the second extracellular loop described above, and a Myc tag on the cytosolic N-terminus of Orai1. Anti-HA fluorescence in an intact cell, therefore, provides an indication of surface expression, whereas anti-Myc fluorescence in a permeabilized cell reflects the entire protein distribution (plasma membrane and cytoplasm). In intact cells expressing WT Orai1 (tagged with HA and Myc), only anti-HA surface labelling was apparent (Figure 2E). In permeabilized cells, anti-myc labelling was distributed through the cytoplasm but excluded from the DAPI-stained nucleus (Figure 2E). Merging of the anti-HA and anti-Myc images showed significant co-localisation at the cell surface but mainly anti-myc staining in the cytoplasm (Figure 2E; three times more WT Orai1 plasmid was used to ensure protein expression was similar to the Orai1–SNPs). The fluorescence ratio of HA-tagged Orai1 in intact cells to myc-tagged Orai1 in permeabilized cells provides an estimate of the fraction of Orai1 channels in the plasma membrane. This was ~ 45% (Figure 2G), in good agreement with a report from CHO cells (Hodeify et al., 2015). Anti-HA staining of S218G–N223S–Orai1 at the cell surface was stronger than for WT Orai1 and there was less intracellular anti-myc labelling (Figures 2F and 2G). Collectively, both cell-surface biotinylation and immunofluorescence studies reveal that Orai1–SNPs are expressed in the plasma membrane more abundantly than WT Orai1, despite similar overall levels of cellular protein expression.

### Impaired Ca^2+^ entry in the presence of Orai1–SNPs is rescued by overexpression of STIM1

There is an optimal STIM1/Orai1 stoichiometry for maximal Ca^2+^ entry (14; 15). Overexpression of Orai1 alone is generally found to reduce store-operated Ca^2+^ entry by introducing a mismatch in the STIM1/Orai1 stoichiometry (15; 23; 30). Too much Orai1–SNP protein in the plasma membrane should also lead to reduced Ca^2+^ entry, provided STIM1 levels have not correspondingly increased, and therefore, there is indeed a mismatch in STIM1/Orai1 stoichiometry. Whereas Orai1–SNP proteins increased significantly over WT Orai1, STIM1 levels remained relatively constant (Figure 1A). Consistent with previous studies, overexpression of Orai1 alone significantly reduced store-operated Ca^2+^ entry (Figure 3A). Expression of each Orai1–SNP also reduced Ca^2+^ influx (Figure 3B and 3C). Since it could not be excluded that the membrane potential was different in cells expressing Orai1–SNPs compared with WT Orai1, control experiments were carried out under conditions where the membrane potential was clamped at 0 mV. This was achieved by exposing cells to high (100 mM) K^+^-containing external solution. In high K^+^-containing solution, the rate of rise of cytosolic Ca^2+^ after readmission of external Ca^2+^ to thapsigargin-treated cells was 55 ± 4% that seen in normal external K^+^. The same difference between Ca^2+^ entry rates with Orai1 and the Orai1 *double variant* was observed when cells were exposed to thapsigargin in high K^+^ solution (Figure 3D and 3F). Similarly, the Ca^2+^ entry rate when S218G–Orai1 was expressed was 62.9 ± 7.2% that of WT Orai1 in high K^+^ solution. The reduced Ca^2+^ entry when Orai1–SNPs were expressed was also not due to increased Ca^2+^ removal, because it remained low when Ba^2+^ was used as a surrogate for Ca^2+^ (Figure 3E and 3F). Ba^2+^ permeates Orai1 channels but is not a substrate for Ca^2+^ATPase pumps (29).

Ca^2+^ entry through Orai1 channels activates the transcription factor NFAT1. Following transfection of a GFP plasmid under an NFAT promoter, stimulation with thapsigargin led to a significant increase in the % of cells expressing GFP (Figure 3G). However, this was significantly reduced when either Orai1–SNP or the double variant was expressed instead (Figure 3G).

We compared Ca^2+^ entry through Orai1–SNPs with loss- and gain-of-function Orai1 mutants. The naturally occurring missense mutant R91W–Orai1 causes severe combined immunodeficiency (9) and fails to evoke any Ca^2+^ influx when expressed in HEK cells (3). The Orai1–SNPs we have characterized, therefore, induce a much milder Ca^2+^ influx phenotype. The gain-of-function mutant P245L–Orai1 associates with Stormorken syndrome (24) and results in a constitutive partially activated channel independent of STIM1 (27). Expression of P245L–Orai1 resulted in an enhanced rate of Ca^2+^ entry (Supplemental Figure 2), compared with WT Orai1.

**Figure 4:**
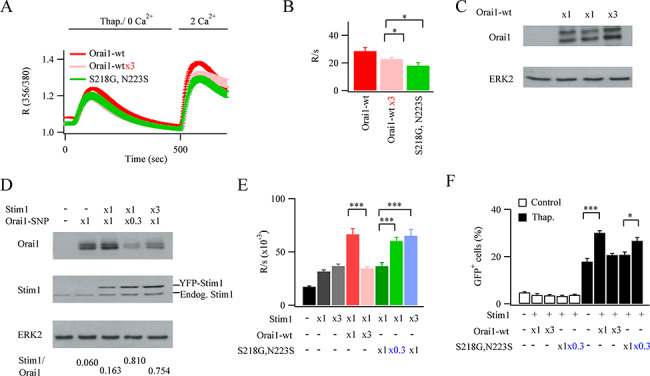
Store-operated Ca^2+^ influx in the presence of Orai1–SNPs can be rescued by increasing STIM1 expression. (**A**) Ca^2+^ release and Ca^2+^ entry are compared between cells expressing WT Orai1 at different levels and S218G–N223S–Orai1. Orai1-wt denotes cells transfected with 200 ng Ora1 plasmid. (**B**) Aggregate data from several experiments as in panel A are summarised. Each bar represents between 15 and 31 cells. (**C**) Western blot compares Orai1 expression following transfection with different amounts of myc-tagged Orai1 plasmid. ×1 denotes 200 ng plasmid. (**D**) Western blot compares levels of expression of STIM1–YFP and myc–Orai1–SNP proteins following transfection with varying amounts of plasmid. The STIM1/Orai1 ratio is denoted below each lane. STIM1 levels reflect the sum of both endogenous and overexpressed protein. For Orai1, the myc tag had little impact on molecular weight. (**E**) The rate of Ca^2+^ entry is compared between experiments in which STIM1, Orai1 and S218G–N223S–Orai1 levels were varied. Each bar denotes 27 to 75 cells. (**F**) Reporter gene expression is compared for varying levels of STIM1, Orai1 and S218G–N223S–Orai1. Each bar denotes between 443 and 785 cells.

If the reduction in store-operated Ca^2+^ entry following expression of Orai1–SNPs is indeed due to a reduced STIM1:Orai1 stoichiometry, due to insufficient STIM1 protein present, then a simple prediction would be that increasing expression of WT Orai1 should mimic the reduction in Ca^2+^ entry seen in the presence of Orai1–SNPs. We tested this by expressing three times the amount of WT Orai1, which results in a similar level of Orai1 protein expression to that seen with Orai1–SNPs (Figure 2B). The rate of store-operated Ca^2+^ entry was now significantly reduced (Figures 4A and 4B) and the reduction was similar to that seen when S218G–N223S–Orai1 was expressed instead. Western blots confirmed that increasing the amount of Orai1 plasmid transfected led to an increase in Orai1 protein expression (Figure 4C).

To test this further, we increased STIM1 expression by transfecting cells with different amounts of YFP–STIM1 plasmid and then evaluated the impact on store-operated Ca^2+^ entry. Increasing the amount of STIM1 plasmid led to an increase in protein expression (Figure 4D). Compared with WT cells, Ca^2+^ influx increased ~ 2-fold when STIM1 was overexpressed by increasing the amount of plasmid used for transfection (Figure 4E). Overexpression of STIM1 and WT Orai1 in a 1:1 plasmid ratio increased Ca^2+^ influx further but this was substantially reduced when the STIM1: WT Orai1 ratio was 1:3 or when STIM1 and S218G–N223S–Orai1 were co-expressed in a 1:1 ratio (western blot shown in Figure 4D and Ca^2+^ influx in Figure 4E). However, when STIM1/S218G–N223S–Orai1 was co-expressed in a 3:1 ratio, Ca^2+^ influx was rescued (Figure 4E; see Figure 4D was western blot). Similarly, when STIM1/S218G–N223S–Orai1 were co-expressed at 1:1/3, Ca^2+^ influx was also rescued (Figure 4E). Similar results were observed with S218G–Orai1. The rate of Ca^2+^ influx increased 2.45 ± 0.2-fold when STIM1 was co-expressed with S218G–Orai1 at a plasmid ratio of 3:1.

Orai1 channel activation of NFAT-driven reporter gene expression was also sensitive to the STIM1:Orai1 ratio (Figure 4F). Overexpression of STIM1 and Orai1 (1:1 ratio) increased gene expression compared with WT cells following stimulation with thapsigargin. An increase in Orai1 levels 3-fold relative to STIM1 or co-expression of STIM1 and S218G–N223S–Orai1 (1,1) reduced gene expression evoked by thapsigargin (Figure 4D). As was the case with Ca^2+^ influx, gene expression could be rescued by expressing STIM1/S218G–N223S–Orai1 in the ratio 1:1/3 (Figure 4F).

### Recycling kinetics show S218G–N223S–Orai1 is endocytosed at a similar rate to WT Orai1

**Figure 5:**
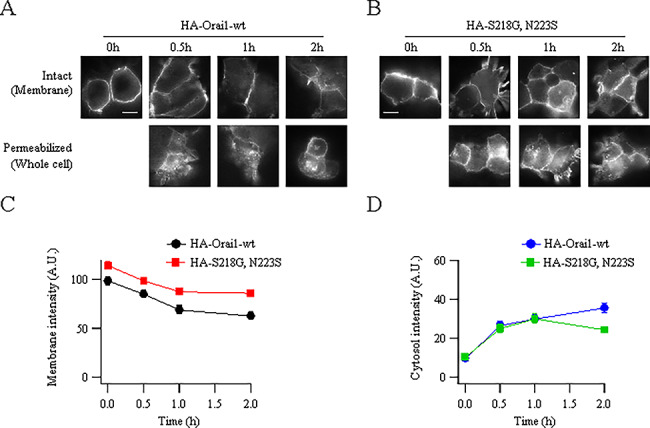
Endocytosis of Orai1 and S218G–N223S–Orai1 are compared. (**A**) Surface and whole cell expression of HA- and myc-tagged Orai1 is shown. Surface expression was analysed in intact cells and whole cell expression after permeabilisation. (**B**) Surface and whole cell expression of S218G–N223S–Orai1 is shown, as in panel A. (**C**) The initial time course of decay of surface labelling is compared between WT and S218G–N223S–Orai1. Each point for the S218G–N223S–Orai1 curve was significantly different from the corresponding control point (*P* < 0.05 at 0 and 0.5 hours; *P* < 0.01 at 1 and 2 hours). (**D**) The time course of the cytosolic increase in myc-tagged Orai1 in permeabilised cells is compared for the two conditions. Between 15 and 20 cells were analysed for each condition and, for each cell, four to six regions of interest were drawn on the membrane or cytosol and averaged. Only the corresponding points at 2 hours were significantly different (*P* < 0.01).

The increase in surface expression of S218G–N223S–Orai1 compared with WT Orai1 is functionally important because it leads to a reduction in store-operated Ca^2+^ entry and Ca^2+^-dependent gene expression. We, therefore, designed experiments to identify the molecular basis for why Orai1–SNPs exhibited increased plasma membrane expression. The increase could reflect reduced endocytosis from the plasma membrane or increased recycling back to the membrane. The finding that inhibition of lysosome function (Supplemental Figure 1) increased Orai1–SNP protein levels slightly suggests that Orai1–SNP proteins are indeed endocytosed. To measure endocytosis more directly, we expressed either HA- and myc-tagged WT Orai1 or HA- and myc-tagged S218G–N223S–Orai1 and then incubated intact cells with anti-HA antibody at 4°C, a temperature than inhibits vesicle cycling to and from the plasma membrane (40). After 1 hour at 4°C, unbound antibody was removed and cells were incubated at 37°C for different times before fixation. Prominent labelling of the cell surface had occurred immediately after antibody removal (Figure 5A; labelled 0 hour) but this declined over time for tagged WT Orai1. The loss of anti-HA surface label first fell relatively steeply with time before declining more slowly (Figure 5C). The slope at early time points provides an estimate of the initial rate of removal of surface protein intensity and this was −29.7 fluorescence units s^−1^ for WT Orai1. Surface membrane intensity of HA-tagged S218G–N223S–Orai1 was higher than that of WT Orai1, as expected from the increased plasma membrane expression, and intensity remained higher at every time point measured (Figure 5B and 5C). Nevertheless, loss of the protein from the cell surface followed a similar initial time course to that of WT Orai1, with a slope of −26.7 fluorescence units s^−1^. These data suggest that both WT Orai1 and S218G–N223S–Orai1 are initially endocytosed at similar rates. Loss of Orai1 proteins from the surface should result initially in an increased accumulation in the cytoplasm. To address this, we used the same protocol as above but, after fixing the cells at different times, we permeabilized the cells to enable myc antibody to access the cytoplasm. Intracellular accumulation of both WT and S218G–N223S–Orai1 were initially similar (Figure 5D; rates of rise of cytoplasmic fluorescence intensity were 33.8 s^−1^ and 29.3 s^−1^, respectively), and were comparable with their initial rates of endocytosis. Thereafter, intracellular accumulation of WT Orai1 diverged from S218–N223S–Orai1 (Figure 5D), reflecting the higher surface expression at later times. These results suggest that both WT Orai1 and S218G–N223S–Orai1 are initially endocytosed with similar kinetics but S218G–N223S–Orai1 recycles back to the plasma membrane more quickly, thereby maintaining high surface expression at the expense of intracellular accumulation and subsequent degradation.

### Orai1 is endocytosed through a dynamin- and flotillin-sensitive pathway

To identify the endocytotic pathway that removes Orai1 from the plasma membrane, we immuno-precipitated WT Orai1–GFP and then used mass spectroscopy to reveal endocytotic proteins that associated with Orai1. Both dynamin, a large GTPase involved in receptor-mediated endocytosis, and flotillin, a membrane protein found involved in vesicle trafficking (22), were found (Supplemental Figure 3). Consistent with a functional role for dynamin, inhibition of dynamin GTPase activity with dynasore significantly reduced Orai1 protein turnover (Supplemental Figure 3), consistent with studies in CHO cells (14).

**Figure 6:**
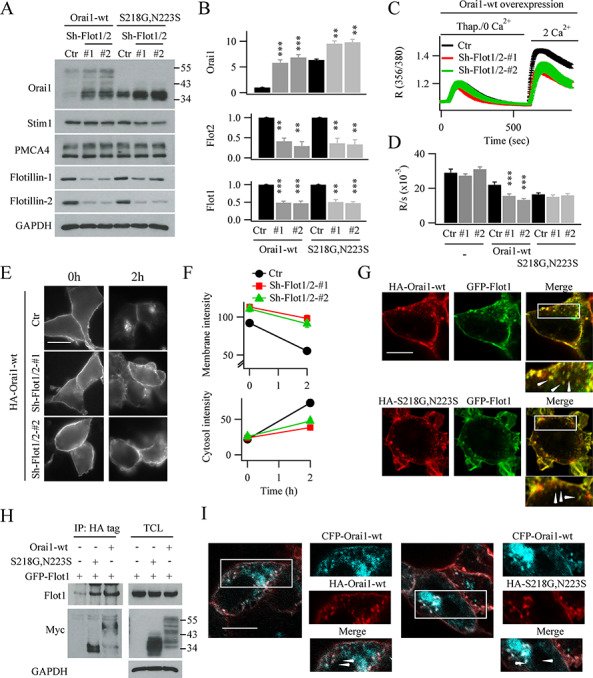
Flotillin regulates Orai1 protein levels. (**A**) Western blot compares knockdown of Flotillin 1 and 2 on expression of WT Orai1, S218G–N223S–Orai1, STIM1, PMCA and flotillin following transfection with two different shRNA pools against flotillin. (**B**) Aggregate data comparing WT Orai1, Orai1–SNP and flotillin expression from experiments as in panel A are shown. (**C**) Store-operated Ca^2+^ entry is compared following overexpression of WT Orai1, for the conditions shown. Each bar is between 28 and 40 cells. (**D**), Bar chart compares data from experiments as in panel C. (**E**) Cell surface and cytsolic staining of HA- and myc-tagged WT Orai1 is shown following knock down of flotillin. (**F**) Graphs compare surface and cytosolic intensities at different times, from experiments as in panel E. For both graphs, Ctr group at 2 hours was significantly different from the others (*P* < 0.01). There was no significant difference between flotillin groups (*P* > 0.1). (**G**) Images compare localisation of HA-tagged WT Orai1 (upper panel) or HA-tagged Orai1–SNP and GFP–flotillin 1. Merged images showed co-localisation on the surface and in intracellular vesicles (region within rectangles have been expanded). (**H**) Immuno-precipitation of HA- and myc-tagged WT Orai1 or HA- and myc-tagged tagged Orai1–SNP with anti-HA antibody reveals the presence of GFP-tagged flotillin. Protein levels in total cell lysate are shown in the right hand gel. Gels shown were taken from the same cell preparation and experiment. (**I**) Images compare distribution of WT CFP–Orai1 with WT HA-tagged Orai1 (left hand panel) with CFP- and HA-tagged S218G–N223S–Orai1 proteins.

To examine the functional relevance of flotillin, we knocked down both isoforms (flotillin 1 and flotillin 2) using two different shRNA smart pools (Figure 6A). Both smart pools reduced flotillin expression by ~ 60% (Figure 6A, aggregate data summarized in Figure 6B). Strikingly, expression of WT Orai1 increased more than 5-fold following knockdown of flotillin. S218G–N223S–Orai1 levels also increased after knockdown of flotillin (Figure 6B), although the relative increase was smaller due to the higher initial levels of Orai1–SNP protein. Expression of STIM1 or the plasma membrane Ca^2+^ATPase pump were unaltered by the reduction in flotillin levels (Figure 6A), demonstrating some specificity towards Orai1.

**Figure 7:**
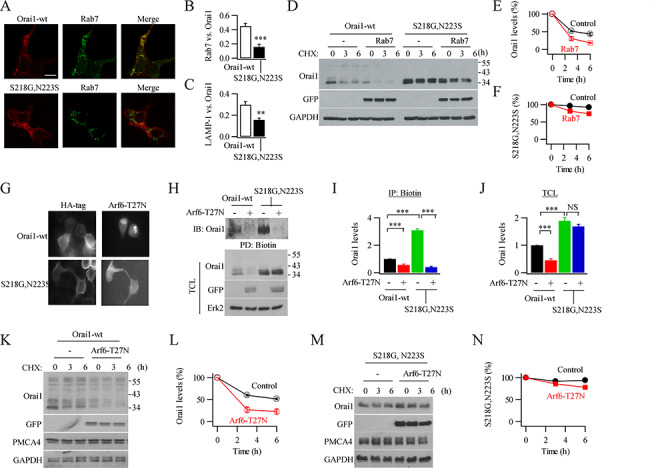
WT Orai1 but not Orai1–SNP accumulates in Rab7-positive late endosomes. (**A**) Images compare distribution of WT Orai1–cherry and Rab–GFP (upper panel) or S218G–N223S–Orai1–cherry and Rab 7–GFP (lower images). (**B**) Bar chart compares co-localisation of Rab-7 with WT Orai1 or double variant. (**C**) Bar chart compares co-localisation of LAMP-1 with WT Orai1 or double variant. (**D**) Western blot shows overexpression of Rab 7 accelerates degradation of wild type Orai1 but not double variant, following treatment with cycloheximide. (**E**) Aggregate data for WT Orai1 from three independent experiments as in panel D are compared. Rab 7 points at 3 hours and 6 hours were significantly different from control (*P* < 0.05 and *P* < 0.01, respectively). (**F**) As in panel E but double variant is shown instead. There was no significant difference between corresponding points at 3 hours (*P* > 0.1) but there was at 6 hours (*P* < 0.01). (**G**) Surface expression of HA-tagged WT Orai1 of double variant is reduced following expression of Arf6–T27N. (**H**) Gel shows surface biotinylation of WT Orai1 or double variant is suppressed by Arf6–T27N. (**I)** Aggregate data from three experiments as in panel H are compared. (**J**) Graph compares WT Orai1 and double variant expression in the total cell lysates used in panel I. (**K**) Expression of Arf6–T27N accelerates the degradation of WT Orai1 protein. (**L**) Graph compares time course of WT Orai1 degradation for the conditions shown. Corresponding points at 3 and 6 hours were significantly different (*P* < 0.001). (**M**) As in panel K but Orai1 double variant is shown. (**N**) Graph compares time course of Orai1–SNP degradation in the absence or presence of Arf6-T27N. Points were not significantly different at 3 hours (*P* > 0.1) but were at 6 hours (*P* < 0.05).

**Figure 8:**
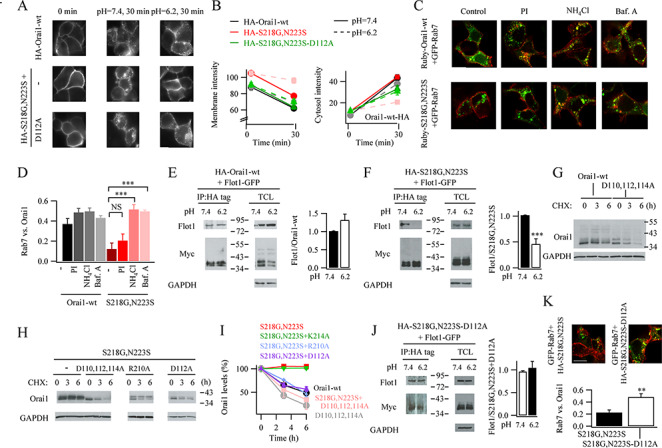
pH regulates Orai1 turnover. (**A**) Images compare surface expression of HA-tagged WT or S218–N223S–Orai1 in different external pH. (**B**) Aggregate data are shown for experiments as in panel A. Left hand graph compares surface expression; right hand graph cytosolic increase. For both graphs, comparing responses at pH 6.2 and 7.4 for the same constructs, the only significant difference was for HA–S218G–N223S. For all other conditions, *P* > 0.1 between pH 6.2 (pink) and 7.4 (red). (**C**) Confocal images compare distribution of Orai1 and Rab 7 for the conditions shown. PI denotes protease inhibitor, used to prevent lysosomal degradation. (**D**) Bar chart compares co-localisation (Pearson’s coefficient) between Rab 7 and Orai1 (WT or Orai1–SNP) for the conditions shown. (**E**) Immunoprecipitation of HA-tagged WT Orai1 reveals presence of flotillin 1, at either pH 7.4 or 6.2. TCL denotes total cell lysate. Aggregate data from 3 independent gels are compared in the bar chart. Difference was not significant (*P* > 0.1). (**F**) As in panel E but S218–N223S–Orai1 was expressed instead. (**G**) Western blot compares time course of degradation of WT Orai1 or D110A–D112A–D114A–Orai1, in cycloheximide-treated cells. (**H**) Blot compares time course of degradation of S218G–N223S–Orai1, D110A–D112A–D114A–Orai1 SNP or D112A–Orai1 SNP. (**I**) Time course of protein degradation is compared for the conditions shown (as in panels G and H). Compared with S218G,N223S graph, S218G,N223S + K214A was not significantly different (*P* > 0.1). S218G,N223S + R210A, S218G,N223S + D112A and S218G,N223S + D110, D112 and D114 were all significantly different from S218G,N223S at 3 and 6 hours (*P* < 0.001, *P* < 0.001 and *P* < 0.01, respectively). (**J**) D112A–Orai1 SNP remains associated with flotillin 1, even in acidic pH. Difference between peaks in bar chart was not significant (*P* > 0.1). (**K**) D112A–Orai1 SNP co-localizes more strongly with Rab 7 than Orai1 SNP.

The increase in Orai1 protein expression after knock down of flotillin should result in a STIM1:Orai1 mismatch and thus impact on Ca^2+^ influx. We, therefore, overexpressed WT Orai1 after knock down of either flotillin-1 or flotillin-2 and found that store-operated Ca^2+^ influx was significantly reduced, compared with overexpression of Orai1 but in the absence of flotillin knockdown (Figures 6C and 6D). We also expressed HA- and myc-tagged Orai1 and monitored surface labelling at different times after removal of anti-HA antibody, as described in Figure 5. In cells expressing HA-tagged WT Orai1, prominent labelling was seen initially but this fell substantially after 2 hours and was accompanied by a corresponding rise in cytosolic protein intensity (Figure 6E and 6F). However, after knockdown of flotillin, surface intensity of HA-tagged WT Orai1 decreased only slightly after 2 hours whilst the cytoplasmic intensity increased only modestly.

These data suggest that Orai1 and S218G–N223S–Orai1 are both initially endocytosed in a flotillin-dependent manner. Consistent with this, significant co-localisation of HA-tagged Orai1 and GFP-tagged flotillin 1 was observed, both at the cell surface and in intracellular structures (Figure 6G, upper panel). Similar results were obtained when HA-tagged S218G–N223S–Orai1 and GFP–flotillin 1 were co-expressed instead (Figure 6G, lower panel). In both cases, Orai1 or Orai1 double variant co-localized with flotillin in intracellular structures. Further evidence for an association between Orai1 proteins and flotillin was provided by co-immunoprecipitation experiments. Anti-HA immuno-precipitation of HA- and myc-tagged WT Orai1 or HA- and myc-tagged S218G–N223S–Orai1 revealed, in both cases, the presence of GFP-tagged flotillin 1 (Figure 6H). Western blots on total cell lysate confirmed similar expression levels of GFP–flotillin 1 and myc-tagged Orai1 (Figure 6H).

Co-expression of CFP–Orai1 and HA-myc–S218G–N223S–Orai1 revealed that both proteins could localize to small intravesicular compartments (Figure 6I), suggesting they occupied the same vesicles.

### WT Orai1 but not S218G–N223S–Orai1 is found in late endosomes

Based on the preceding results, we hypothesized that, after initial endocytosis with flotillin into early endosomes, WT Orai1 is targeted towards the late endosome/lysosomal degradation system whereas S218G–N223S–Orai1 is directed instead towards the recycling pathway. The small GTP-binding protein Rab 7 is involved in early to late endosome traffic and has been localized to late endosomes and lysosomes (41). Following co-expression of either Ora1–cherry or S218G–N223S–Orai1–cherry with Rab 7–GFP (Figure 7A), we found significantly more co-localisation between WT Orai1 and Rab 7 than was the case between S218G-N223S–Orai1 and Rab 7 (Figure 7B). Co-staining of WT Orai1 or S218G–N223S–Orai1 with LAMP-1, a marker for lysosomes, revealed significantly more co-localisation of LAMP1 with WT Orai1 than with S218G–N223S–Orai1 (Figure 7C). After expression of either Rab 7–GFP and myc-tagged Orai1 (control cells) or Rab 7–GFP and myc-tagged S218G–N223S–Orai1, we blocked protein synthesis by treating cells with cycloheximide. Whereas ~ 50% of WT Orai1 in the control group was degraded within 6 hours, this fraction was increased to ~ 75% after expression of Rab 7 (Figure 7D and 7E). By contrast, S218G–N223S–Orai1 protein was relatively stable (Figure 7D and 7F), despite expression of Rab 7–GFP to levels similar to the WT Orai1 control group.

Proteins in early endosomes have one of two fates: they can progress, through a Rab 7-dependent pathway to late endosomes and then lysosomes or they can be recycled back to the plasma membrane either directly through a Rab 4-dependent fast cycling route or indirectly via a slower Rab 11-dependent recycling endosome pathway (37; 38; 39). Recycling of both types of endosome to the plasma membrane requires the small GTP-binding protein Arf 6(5; 31). Expression of a dominant negative Arf 6 protein (T27N–Arf 6) that blocks endosomal recycling to the plasma membrane, therefore, should lead to accumulation of intracellular protein (D’Souza-Schorey et al., 1995). After treatment with cycloheximide, T27N–Arf 6-expressing cells showed reduced surface expression of both HA-tagged WT Orai1 and HA-tagged S218G–N223S–Orai1 (Figure 7G). For Orai1–SNP, more protein was in the cytosol than for WT Orai1 (Figure 7A), consistent with less degradation of Orai1–SNP.

If T27N–Arf 6 was indeed blocking recycling, then there should be less Orai1 protein in the plasma membrane. Following expression T27N–Arf 6, both biotinylated WT Orai1 and S218G–N223S–Orai1 protein levels in the membrane were low (Figure 7H and 7I). However, analysis of total cell lysate showed a significant fraction of WT Orai1 protein had been degraded in the presence of T27N–Arf 6, whereas S218G–N223S–Orai1 protein remained at high levels (Figure 7H and 7J). Consistent with this, following expression of Arf6–T27N, WT Orai1 was degraded relatively quickly after cycloheximide treatment with 75% of protein lost by 3 hours (Figure 7K and 7L). Loss of Orai1–SNP protein was much less, falling by 20% after 6 hours (Figure 7M and 7N).

These experiments show that recycling of WT Orai1 and S218G–N223S–Orai1 back to the plasma membrane both require Arf 6. However, when the recycling process is impaired, endocytosed WT Orai1 proteins proceed to the lysosomal degradation pathway. However, intracellular S218G–N223S–Orai1 protein is not targeted for degradation and, therefore, is retained within the cytoplasm in recycling endosomes. Accumulation of HA-tagged Orai1–SNP in the cytosol for tens of minutes (Figure 5D) suggests endosome recycling is not via the fast pathway but by the Rab 11-dependent slower route. If S218G–N223S–Orai1 accumulates in slow recycling endosomes in T27N–Arf 6-expressing cells, the protein should co-localize relatively strongly with Rab 11-positive but poorly with Rab 7-positive endosomes. Conversely, WT Orai1 should co-localize relatively well with Rab 7-positive but not Rab 11-positive vesicles. The data in Supplemental Figure 4 confirm this prediction. The distribution of WT Orai1 overlapped poorly with Rab 11 but strongly with Rab 7, in T27N–Arf 6-expressing cells. By contrast, S218G–N223S–Orai1 co-localized considerably better with Rab 11 than with Rab 7.

Flotillin is often found in late endosomes, suggesting it might help regulate cargo trafficking to the lysosomal degradation pathway. Since WT Orai1 is transported to lysosomes through late endosomes, whereas S218G–N223A–Orai1 is not, we asked whether flotillin was also found in late endosomes but not recycling endosomes. To address this, we co-expressed mcherry-flotillin 1 with Rab 7 or Rab 11 (both GFP-tagged). There was resolvable co-localisation of flotillin 1 with Rab 7 in intracellular vesicular structures (Supplemental Figure 5), but much less between flotillin 1 and Rab 11.

### Orai1–flotillin interaction is sensitive to ambient pH

Since flotillin is involved in the endocytosis of both WT Orai1 and Orai1–SNPs and accumulates within Rab 7-labelled late endosomes (19), we reasoned that Orai1–SNP was able to separate from flotillin in early endosomes, whereas WT Orai1 was not, providing a mechanism for Orai1–SNPs to escape the degradation pathway. Because the Orai1–SNPs are on the second extracellular loop, they would face inside the endosome. The pH gradually decreases from about 6.5 in early endosomes to close to 5.5 in late endosomes (11). We, therefore, hypothesized that low pH might enable Orai1–SNPs but not WT Orai1 to separate from flotillin. To examine this, we expressed HA-tagged Orai1 or HA-tagged Orai1 double variant and measured the extent of membrane staining 30 minutes after exposure to external solution of either pH 7.4 or 6.2. For HA-tagged WT Orai1, membrane staining fell~ 40%, and this was unaffected by external pH (Figure 8A and 8B). Cytosolic Orai1 rose after 30 minutes, consistent with loss of surface expression (Figure 8B). A similar decrease in S218G–N223S–Orai1 surface expression was seen at pH 7.4 (Figure 8A and 8B) but, at pH 6.2, surface expression was maintained (Figure 8A and 8B). Raising pH within an endosome should prevent separation of flotillin and Orai1–SNP and, therefore, result in the appearance of Orai1–SNP in Rab 7-containing late endosomes. Following co-expression of ruby-tagged WT Orai1 and GFP–Rab 7, we found partial co-localisation of the two proteins (Figure 8C and 8D), and this increased slightly following exposure to NH_4_Cl or bafilomycin (Figure 8C and 8D). Co-expression of ruby-tagged S218G–N223S–Orai1 and Rab 7–GFP showed significantly less co-localisation (Figure 8C and 8D) but this increased substantially in the presence of either NH_4_Cl or bafilomycin and reached a level similar to that seen with WT Orai1 under the same conditions (Figure 8C and 8D).

We investigated pH-dependent association of flotillin and Orai1 by carrying out co-immunoprecipitation experiments at different ambient pH following co-expression of HA- and myc-tagged Orai1 and GFP–flotillin. Following pull down of HA-tagged WT Orai1 at pH 7.4, GFP was detected and the amount of GFP present increased slightly at pH 6.2 (Figure 8E). As was the case with WT Orai1, GFP was detected after pull down of HA-and myc-tagged S218G–N223S–Orai1 at pH 7.4 (Figure 8F). However, significantly less GFP was found at pH 6.2, consistent with separation of Orai1–SNP and flotillin at acidic pH levels found in endosomes (Figure 8F).

### Electrostatic interactions between extracellular loops 1 and 2 increases Orai1 turnover

We looked for amino acids in the second extracellular loop that could be affected by pH. In human Orai1, this loop is devoid of histidine residues which could have imparted pH sensitivity. However, there are three closely spaced negatively charged aspartates (D110, D112 and D114) within the first extracellular loop of Orai1. Mutating both D110 and D112 prevents pH-dependent regulation of Orai1 (1).

There are two positively charged amino acids on the second extracellular loop of Orai1, R210 and K214 which both lie close to the SNPs. Molecular simulation dynamics have found that R210 interacts electrostatically with D110, D112 and D114 on the first extracellular loop of Orai1, with a favoured interaction with D112 (Frischauf et al., 2015).

To test for electrostatic interactions, we mutated all three aspartates to alanines in loop 1 of both WT Orai1 (Figure 8G) and Orai1 double variant (referred to as 3D–3A–S218G–N223S–Orai1; Figure 8H). For both mutant channels, degradation was considerably faster than for either WT Orai1 or Orai1 double variant (Figure 8I). The time course of loss of 3D–3A–S218G–N223S–Orai1 was similar to that seen for WT Orai1, suggesting the asparate residues reduced Orai1 turnover. We also mutated the positively charged R210 and K214 residues in the second extracellular loop of S218G–N223S–Orai1 to alanines. Protein loss was accelerated after expression of R210A–S218G–N223S–Orai1 but not K214A–S218G–N223S–Orai1 (Figure 8H and 8I).

Because R210 interacts mainly with D112, we mutated D112 to alanine, leaving D110 and D114 intact. Following expression of D112A–S218G–N223S–Orai1, protein degradation in cycloheximide-treated cells proceeded considerably more quickly than S218G–N223S–Orai1 (Figure 8H and 8I). The time course of loss of D112A–S218G–N223S–Orai1 was similar to that of 3D–A–S218G–N223S–Orai1, suggesting a major role for D112.

Unlike S218G–N223S–Orai1, which separates from flotillin 1 at pH 6.2, D112A–S218G–N223S–Orai1 retained interaction (Figure 8J). Co-expression of D112A–S218G–N223S–Orai1 with Rab 7–GFP revealed considerably more co-localisation of the two proteins than was the case with Rab 7–GFP and S218G–N223S–Orai1 (Figure 7K), demonstrating that D112A–S218G–N223S–Orai1 now accumulated in late endosomes.

## Discussion

Flux through an ion channel depends not only on unitary conductance and open probability but also on the number of available channels (12). One way to increase the number of available channels is through an enhancement in the rate of insertion into, or a reduction in removal from, the plasma membrane. Many ion channels are rapidly endocytosed and then face one of two fates: they are either recycled back to the membrane or are targeted to lysosomes for degradation.

Our data show that WT human Orai1, two common human Orai1–SNPs and the double variant are endocytosed in a dynamin- and flotillin-sensitive process and initially share the same intracellular vesicles. Thereafter, WT Orai1 is shunted to Rab 7-positive late endosomes and then lysosomes where the channels are degraded. Orai1–SNPs escape from early endosomes to enter Rab 11-positive recycling endosomes, which are then returned to the plasma membrane through an Arf 6-sensitive endosome-plasma membrane fusion process. How proteins are separated within early endosomes and then targeted for degradation or recycling is not entirely clear but, in some cases, specific amino acid motifs on cytosolic portions of recycled proteins are thought to be important in dictating the trafficking process. Such signal sequences include a di-leucine motif, YXX-phi type motifs where Y is tyrosine, X is any amino acid and phi is an amino acid with a large bulky hydrophobic side chain, a GDAY motif and a di-leucine motif with a closely spaced cluster of acidic amino acids (16; 28). However, the cytosolic regions of human Orai1 do not contain any of these motifs. Furthermore, the SNPs localize to the second extracellular loop of Orai1, which faces the lumen of early endosomes. Therefore, unless there is a marked change in Orai1 structure, it is difficult to envisage how cytosolic recycling proteins will identify Orai1–SNPS. Endosomes are acidic and we have found that, at a pH that mimics the acidic environment of the early endosome, Orai1–SNPs are able to dissociate from flotillin whereas WT Orai1 protein cannot. WT Orai1 proceeds together with flotillin to late endosomes and lysosomes where they are degraded. Our results, therefore, identify pH of the early endosome as a new signal that helps traffic proteins to different endosomes, and this is accomplished through pH-dependent control of the association of cargo with regulatory proteins such as flotillin.

The difference in protein turnover between WT Orai1 and the Orai1–SNPs could involve a transient electrostatic interaction between D110/D112/D114 in extracellular loop 1 and R210 in extracellular loop 2, first identified in molecular dynamic simulations (10). Our mutagenesis studies identify a major role for D112. An Orai1 channel in which D110 and D112 were both mutated was no longer blocked by external acidification (1). Protonation of D112 in the acidic environment of the endosome would reduce electrostatic interaction between D112 and R210. This looser structure may interact better with flotillin, resulting in transport to late endosomes. By contrast, Orai1–SNP might maintain some association between D112 and R210, reducing interaction with flotillin. Alternatively, flotillin could interact with Orai1 in a pH-dependent manner with the Orai1–SNPs reducing the affinity for flotillin. It is interesting to note that insertion of an HA tag 9 amino acids long after P214 in the second extracellular loop of Orai1 did not alter channel turnover compared with non-tagged WT Orai1. Similarly, insertion of the HA tag did not alter the slower turnover of Orai1–SNPs. These findings can be explained by the observation, drawn from a detailed molecular simulation dynamics study, that the second extracellular loop of Orai1 is, by far, the most flexible domain of the Orai1 channel (10).

A recent study has identified a major role for the intracellular loop in Orai1 involving amino acids 157–167 in regulation of channel trafficking (13). This region was found to interact with the chaperonin-containing TCP-1 complex (T-complex protein) chaperonin complex to modulate Orai1 endocytosis. Knockdown of the complex or expression of an Orai1 construct in which amino acids 157–167 had been scrambled both increased Orai1 plasma membrane residence. It is possible that the TCP-1 complex interacts less strongly with Orai1–SNPs compared with Orai1 or that the interaction is enhanced by the association of flotillin. The effect of the complex on Orai1–SNP trafficking is currently under investigation.

Altered ion channel trafficking is associated with various diseases. The most common mutation in CFTR (ΔF508) generates a Cl^−^ channel that is not transported to the plasma membrane, leading to cystic fibrosis (17). Nonsense mutations in Ether-A-Go-Go K^+^ channels result in impaired channel trafficking and this is associated with long QT syndrome type 2, which can result in sudden cardiac arrest (36). Mutations in the PY motif of β and γ subunits of the epithelial Na^+^ channel (ENaC) result in an inability of E3 ligase Ned4.2 to recognize the channels and mark them for degradation, resulting in high levels of ENaC accumulation in the plasma membrane, causing Liddle syndrome (21). The accumulation of Orai1–SNPs in the plasma membrane might, like the PY mutations in ENaC, cause a gain-of-function phenotype. However, we found that Ca^2+^ influx was reduced by the increase in Orai1 channel expression. This arose from a mismatch between the levels of STIM1 and Orai1 and increasing STIM1 expression rescued the defect in Ca^2+^ entry. Although mechanisms regulating transcription of STIM1 have been described (35), control of STIM protein turnover has been hitherto unexplored but could be an important factor in the long-term control of store-operated Ca^2+^ entry.

Our results reveal an interesting difference between N-linked glycosylated and non-glycosylated Orai1 channels. Only non-glycosylated WT Orai1 was degraded within the time frame of our experiments; glycosylated WT Orai1 was relatively stable. Other ion channels exhibit greater stability in the plasma membrane when glycosylated including HERK K^+^ and Na^+^ channels (32; 42). Whether glycosylated Orai1 is endocytosed like WT Orai1 but mimics Orai1–SNPs in escaping to recycling endosomes or is simply retained for longer in the plasma membrane will be an interesting question to address in the future. Jurkat T cells stably expressing HA-tagged WT Orai1 or HA-tagged N223A–Orai1 showed slightly larger Ca^2+^ influx for the non-glycosylated channel (1). In our experiments, we observed reduced Ca^2+^ entry following expression of N223A–Orai1. This difference likely arises from the fact that we used transient transfection, which produces stronger protein expression. Higher levels of N223A–Orai1 in our experiments would exaggerate the mismatch in STIM:Orai1 levels and lead to reduced Ca^2+^ influx.

Our data show that ~ 50% of WT Orai1 channels are endocytosed from the plasma membrane with a half-time of ~ 2 hours. We also find that 50% of WT Orai1 is degraded after 6 hours. Therefore, there is a marked temporal dissociation between endocytosis and degradation. Degradation of proteins in lysosomes proceeds rapidly. Ribonuclease (molecular weight of ~ 12.4 kDa) is degraded in lysosomes with a half-time of ~ 11 minutes (6). Assuming linear scaling, non-glycosylated Orai1 would be degraded within lysosomes after ~ 60 minutes, which is faster than the duration of endocytosed Orai1 in the cytosol. This raises the question of why endocytosed Orai1 is maintained in the cytosol for so long before degradation. One possibility is that Orai1 contained in cytosolic vesicles acts as a reservoir for plasma membrane channels, inserting into the membrane upon stimulation. This would be analogous to insertion of stored aquaporin-2 water channels into the apical pole of renal collecting duct epithelial cells in response to vasopressin (25). An intriguing additional possibility is that intracellular vesicles of Orai1 function as mobile Ca^2+^ stores, shuttling through the cytosol. Vesicular Orai1 would still be gated by STIM1 and, when activated, would release Ca^2+^ from the endosome into the cytosol. Intracellular Ca^2+^ release driven by STIM1–Orai1 interaction from phagosomes (26) and secretory granules (7) has been demonstrated. As STIM proteins are primarily expressed in the ER, Orai1 endosomes may function as sources of trigger Ca^2+^ regulating Ca^2+^ − dependent processes proximal to the ER.

## Materials and methods

### Cell culture and treatment

HEK293T were purchased from ATCC (via the United Kingdom supplier LGC). HEK239T cells were cultured in Dulbecco’s modified Eagle’s medium (DMEM) (Thermo Scientific). Medium was supplemented with 10% fetal bovine serum and 1% penicillin-streptomycin. After transfection with Lipofectamine 2000 (Invitrogen), HEK293T cells were incubated in media without penicillin-streptomycin. Experiments were then carried out 24 to 48 hours after transfection.

The following drugs were used: cycloheximide (100 μg/ml; Cambridge Bioscience), Dynasore (80 μM; Sigma), NH_4_Cl (20 mM; Sigma), Bafilomycin a1 (100 μM; Sigma), BTP2 (10 μM, gift from Dr Daniel Bakowski, Calcico Therapeutics).

### Plasmid constructs

The myc-tagged mutant Orai1 constructs S218G, N223S, D110A/D112A/D114A, R210A, K214A, D112C and R210C were generated using the QuikChange Lightning site-directed mutagenesis kit (Agilent Technologies) with the following primers:

**Table:** 

S218G	Forward: 5′-GCCCCCCGCC**GGT**GGCGCAGC-3′
	Reverse: 5′-GCTGCGCC**ACC**GGCGGGGGGC-3′
N223S	Forward: 5′-GGCGCAGCAGCC**AGC**GTCAGCACCA-3′
	Reverse: 5′-TGGTGCTGAC**GCT**GGCTGCTGCGCC-3′
D110A/D112A/D114A	Forward: 5′-GTGCAGCTG**GCC**GCT**GCC**CAC**GCC**TACCCACCG-3′
	Reverse: 5′-CGGTGGGTA**GGC**GTG**GGC**AGC**GGC**CAGCTGCAC-3′
R210A	Forward: 5′-GCAGCCAGGCCAGCCA**GCG**CCCACCAG-3′
	Reverse: 5′-CTGGTGGG**CGC**TGGCTGGCCTGGCTGC-3′
K214A	Forward: 5′-GGCCCACCAGC**GCG**CCCCCCGCCG-3′
	Reverse: 5′-CGGCGGGGGG**CGC**GCTGGTGGGCC-3′
D112C	Forward: 5′-AGGTGCAGCTGGACGCT**TGC**CACGACTACCC-3′
	Reverse: 5′-GGGTAGTCGTG**GCA**AGCGTCCAGCTGCACCT-3′
R210C	Forward: 5′-CAGCCAGGCCAGCCA**TGC**CCCACCAGC -3′
	Reverse: 5′-GCTGGTGGG**GCA**TGGCTGGCCTGGCTG-3′

HA- and myc-tagged Orai1 constructs were generated by using overlapping PCR with the following primers:

**Table:** 

EcoRI–Orai1-Forward	5′-GGCCCGAATTCGGATGCATC-3′
HA-Orai1-Reverse	5′-AGCGTAATCTGGAACATCGTATGGGTACTTGCTGGTGGGCCTTGGCT-3′
HA-Orai1–S218-Forward (2)	5′-TACCCATACGATGTTCCAGATTACGCTCCCCCCGCCAGTGGCGCAGC-3′
HA-Orai1–G218-Forward (2)	5′-TACCCATACGATGTTCCAGATTACGCTCCCCCCGCCGGTGGCGCAGC-3′
NheI–Orai1-Reverse (2)	5′-GCCAAGCTAGCATGCCTGCA-3′

**Table:** 

Ruby–Orai1 (or Orai1–S218G, N223S)	Forward: 5′-CTCAAGCTTCGAATTCG**G**ATGCATCCGGAG-3′
	Reverse: 5′- TAGATCCGGTGGATCCCCGCGGCCGCGGT-3′
Single base deletion	Forward: 5′-TCCGGATGCATCGAATTCGAAG-3′
	Reverse: 5′-CTTCGAATTCGATGCATCCGGA-3′ (Use to remove the extra base between Ruby and Orai1)

**Table:** 

vRuby–Orai1 (or vRuby–S218G, N223S)	Forward: 5′-ACACCGACTCTACTAGAGGATCCACCATGGTGTCTAAGGGCGA-3′
	Reverse: 5′-CCTCTAGACTCGAGCGGCCGCACCCTAGGCATAGTGGCTGC-3′

Ruby–Orai1-WT and Ruby–S218G–N223S–Orai1 were generated by inserting the Orai1-WT or S218G–N223S–Orai1 into the mRuby2-C1 plasmid (Addgene 54768) between EcoRI and BamHI (New England BioLabs) sites by using the In-Fusion Cloning Plus CE kit (Clontech). Both Orai1-WT and S218G-N223S-Orai1 were amplified by PCR amplification with the following primers:

To generate Orai1 in viruses, Ruby–Orai1-WT or Ruby–S218G–N223S–Orai1 were reconstructed into pLex plasmid, between BamHI and NotI (New England BioLabs) sites, by using the following primers:

The Arf6–T27N–CFP construct was purchased from Addgene (11386; donated by Dr Joel Swanson). Flotillin 1–GFP and flotillin 2–GFP were kind gifts from Prof. Ben Nichols (MRC LMB, University of Cambridge, UK). We replaced GFP with mCherry to make a flotillin 1–mCherry construct, and ligation was carried out with the In-Fusion Cloning Plus CE kit (Clontech). Primers used to amplify mCherry are listed below:

**Table:** 

BamHI–mCherry–NotI	Forward: 5′-TACCGCGGGCCCGGGATCCAATGGTGAGCAAGGGCGAGGAG-3′
	Reverse: 5′-TTATGATCTAGAGTCGCGGCCGCTCTACTTGTACAGCTCGTCCATGCC-3′

Rab 7–GFP and Rab 11–GFP were kind gifts from Dr Adam Grieve (Department of Pathology, Oxford, UK).

Two oligonucleotides containing the targeting sequences for flotillin 1 or flotillin 2 are listed below. The annealing of two oligos, 500 μM of each oligos in total 20 μL annealing buffer (10 mM Tris, 1 mM EDTA, 50 mM NaCl, pH = 8.0), was carried out by a gradual decrease in temperature from 98°C to 18°C (~1 °C per sec). After gel purification, oligos were ligated into the pre-cut pLKO TRC005 plasmid within the KpnI and EcoRI (New England BioLabs) sites by T4 DNA ligase (Thermo Scientific).

ShRNA target sequences:

**Table:** 

Control shRNA-top	5′-cggTAAGGCTATGAAGAGATACctcgagGTATCTCTTCATAGCCTTAtttttg-3′
Control shRNA-bottom	5′-aattcaaaaaTAAGGCTATGAAGAGATACctcgagGTATCTCTTCATAGCCTTAccggtac-3′

Flot1-shRNA 1-top	5′-cggGCAGAGAAGTCCCAACTAATTctcgagAATTAGTTGGGACTTCTCTGCtttttg-3′
Flot1-shRNA 1-bottom	5′-aattcaaaaaGCAGAGAAGTCCCAACTAATTctcgagAATTAGTTGGGACTTCTCTGCccggtac-3′
Flot1-shRNA 2-top	5′-cggCTCTCCTTGCCAAATAGTTTGctcgagCAAACTATTTGGCAAGGAGAGtttttg-3′
Flot1-shRNA 2-bottom	5′-aattcaaaaaCTCTCCTTGCCAAATAGTTTGctcgagCAAACTATTTGGCAAGGAGAGccggtac-3′
Flot1–shRNA 3-top	5′-cggGGAAGTACTGGACATTCTAACctcgagGTTAGAATGTCCAGTACTTCCtttttg-3′
Flot1–shRNA 3-bottom	5′-aattcaaaaaGGAAGTACTGGACATTCTAACctcgagGTTAGAATGTCCAGTACTTCCccggtac-3′

Flot2-shRNA A-top	5′-cggCGTGTATGACAAAGTGGACTActcgagTAGTCCACTTTGTCATACACGttttg-3′
Flot2-shRNA A-bottom	5′-aattcaaaaaCGTGTATGACAAAGTGGACTActcgagTAGTCCACTTTGTCATACACGccggtac-3′
Flot2-shRNA B-top	5′-cggGAAGAGATTGAGATTGAGGTTctcgagAACCTCAATCTCAATCTCTTCtttttg-3′
Flot2-shRNA B-bottom	5′-aattcaaaaaGAAGAGATTGAGATTGAGGTTctcgagAACCTCAATCTCAATCTCTTCccggtac-3′
Flot2-shRNA C-top	5′-cggGAGACAACAGTAAGGTCACATctcgagATGTGACCTTACTGTTGTCTCtttttg-3′
Flot2-shRNA C-bottom	5′-aattcaaaaaGAGACAACAGTAAGGTCACATctcgagATGTGACCTTACTGTTGTCTCccggtac-3′

### Quantitative RT-PCR

Cells were transfected with 200 ng of Orai1, S218G–Orai1, N223S–Orai1 or double variant or 600 ng of WT Orai1 plasmids using lipofectamine 2000. After 24 hours, RNA was extracted using an RNeasy Mini Kit (QIAGEN) and quantified spectrophotometrically by absorbance at 260 nm. Total RNA (1 μg) was reverse-transcribed using the iScript™ cDNA Synthesis Kit (Bio-Rad), according to the manufacturer’s instructions. Quantitative real-time RT-PCR was processed with cDNA, Taqman Universal PCR Master mix (Applied Biosystems), H_2_O and specific primers for Taqman Gene Expression Assays (Hs00385627_m1 for human orai1; Hs01060665_g1 for human actin). The samples were loaded onto 96-well and analysed by the ABI Prism 7000 Sequence Detection System software (Applied Biosystems). The qPCR conditions were as follows: 2 minutes at 50°C, 10 minutes at 95°C, followed by 40 cycles of 15 seconds at 95°C and 1 minute at 60°C. For quantification, the relative quantity of samples was calculated according the comparative △C_t_ method and normalized to β-actin.

### Cytosolic Ca^2+^ imaging

HEK239T cells were loaded with Fura 2 following incubation in 1 μM Fura 2-AM in external solution (145 mM NaCl, 2.8 mM KCl, 2 mM CaCl_2_, 2 mM MgCl_2_, 10 mM D-glucose, 10 mM HEPES, pH 7.4) for 40 minutes in the dark, and then cells were washed and incubated in external solution for another 15 minutes for full de-esterification.

Ca^2+^-free solution was comprised of 145 mM NaCl, 2.8 mM KCl, 2 mM MgCl_2_, 10 mM D-glucose, 10 mM HEPES, 0.1 mM EGTA, pH 7.4. High K^+^ solution contained 100 mM KCl, 45 mM NaCl, 2 mM MgCl_2_, 10 mM D-glucose, 10 mM HEPES, 2 mM CaCl_2_. For high K^+^, Ca^2+^-free solution, CaCl_2_ was not present and 0.1 mM EGTA added. Cells were alternately excited at 356 and 380 nm, and signals were acquired every 2 seconds. Calcium signals are represented by the 356 nm/380 nm ratio (R). All the images were analysed by using IGOR Pro software.

### NFAT gene expression

HEK239T cells were co-transfected with either WT Orai1, Orai1–SNPs or the double variant together with an NFAT promoter-driven EGFP-based reporter plasmid. 24 hours after transfection, cells were stimulated with 500 nM thapsigargin in medium for 4 minutes and then thapsigargin was washed and cells incubated with medium for another 24 hours. The percentage of cells that were positive for GFP was then measured.

### Western blotting, immunoprecipitation, and pull down.

Cell lysates were harvested with 1% Triton X-100 lysis buffer (150 mM NaCl, 20 mM Tris–HCl, pH = 7.5, 100 μM sodium orthovanadate (Na_3_VO_4_), 100 μM phenylmethanesulfonyl fluoride (PMSF), and 1× protease inhibitor cocktail (Sigma)). Protein concentrations were assessed by Lowry assay; 10–30 μg of protein lysates were subjected to SDS-PAGE and followed by immunoblotting with specific primary antibodies. Antibodies against STIM1 (D88E10), GFP (D5.1), HA tag (C29F4) and Myc tag (71D10) were purchased from Cell Signaling. Antibodies against Orai1 (O8264) and GAPDH (G8795) were purchased from Sigma. Antibodies against Flotillin-1 (A6220), Flotillin-2 (A6590), or PMCA (JA9) were purchased from ABclonal or Novus Biologicals, respectively. Antibody against Erk2 (C-14) was purchased from Santa Cruz Biotechnology. Erk2 or GAPDH was used as a loading control. Quantification results were represented after normalize with the loading control in each time of experiment.

For immunoprecipitation, 500 μg of protein lysates from HEK239T cells was used. Cells were first co-transfected with HA-inserted Orai1 and Flotillin-1–GFP and, 24 hours later, cells were lysed in 500 μL 1% Triton X-100 lysis buffer. Cell lysates were incubated with anti-HA antibody and 50 μL of protein A Sepharose (GE Healthcare) at 4°C for 2 hours. Beads were spun down and washed three times with ice-cold PBS. The protein levels of Flotillin-1–GFP and Orai1 were assessed with anti-GFP and myc antibodies, respectively. GFP–Trap (ChromoTek) was used to pull down CFP-tagged Orai1 following the manufacturer’s protocol.

### Biotinylation for cell surface proteins

Twenty-four hours after transfection with different Orai1 constructs (see text), HEK293T cells were washed with ice-cold PBS (pH = 8) to remove culture media. Cells were then incubated with 500 μM Sulfo-NHS-Biotin (Thermo Scientific) in PBS at 4°C for 1 hour, followed by three washes in PBS containing 100 μM glycine to quench and remove excess biotin. Cell lysates were harvested with 1% Triton X-100 lysis buffer. 0.5 μg/μL of 500 μL of total cell lysates were incubated with 20 μL of streptavidin-sepharose beads (Cell Signaling) at 4°C for 1 hour to pull down the biotin-labeled cell surface protein. The protein levels of Orai1 were then assessed with antibody after running in an 8% SDS-PAGE.

### Immunocytochemistry and confocal image

Cells were fixed at various times (see text) with 4% paraformaldehyde and permeabilized with 0.5% Triton X-100. Cells were then incubated with blocking solution (Thermo Scientific) for 1 hour at room temperature followed by incubation with primary antibodies against HA tag (C29F4) or Myc tag (71D10).

For measurements of surface expression, cells were washed with ice-cold PBS and then incubated with anti-HA antibody (1500) at 4°C for 1 hour. Cells were washed again with media to remove un-bound antibody, and then incubated with media at 37°C for the indicated time periods. To chase the endocytosis of labeled Orai1 under different pH solutions, cells were incubated in PBS with different pH values at 37°C for 30 min. A Nikon epifluorescence microscope with a 100× oil immersion lens was used. Some images were taken with a FV-1000 (Olympus) confocal microscope with a 60× oil immersion lens. The fluorescence intensity and co-localisation coefficient from two channels represented by Pearson’s *R* value were assessed by Image J software.

### Statistical analysis

All results were expressed as means ± SEM. Two-tailed Student t test was used to compare differences between two groups in all the experiments. GraphPad Prism was used, and statistical significance was set at a *P* value of < 0.05. In all the graphs, *, **, and *** denote *P* value < 0.05, 0.01, and 0.001, respectively.

## Funding

This work was supported by an MRC Programme Grant (LO1047X) to A.B.P.

## Conflict of Interest Statement.

The authors state they have no conflicts of interest.
